# Leptin Increases Expression of 5-HT_2B_ Receptors in Astrocytes Thus Enhancing Action of Fluoxetine on the Depressive Behavior Induced by Sleep Deprivation

**DOI:** 10.3389/fpsyt.2018.00734

**Published:** 2019-01-07

**Authors:** Xiaowei Li, Shanshan Liang, Zexiong Li, Shuai Li, Maosheng Xia, Alexei Verkhratsky, Baoman Li

**Affiliations:** ^1^Laboratory Teaching Center, School of Forensic Medicine, China Medical University, Shenyang, China; ^2^Department of Orthopaedics, The First Hospital, China Medical University, Shenyang, China; ^3^Faculty of Life Science, The University of Manchester, Manchester, United Kingdom; ^4^Achucarro Center for Neuroscience, IKERBASQUE, Basque Foundation for Science, Bilbao, Spain

**Keywords:** sleep deprivation, 5-HT_2B_ receptors, astrocytes, fluoxetine, leptin, NLRP3 inflammasomes, BDNF

## Abstract

The long-lasting loss of sleep is a generally acknowledged risk factor for the occurrence of major depressive disorder (MDD), whereas sleep abnormalities being a key clinic symptom of the MDD. In our previous work, we demonstrated that the sleep deprivation (SD) stimulates activation of nucleotide-binding domain and leucine-rich repeat protein-3 (NLRP3) inflammasomes as well as the release of IL-1β and IL-18 from astrocytes. However, the underlying mechanism connecting SD and MDD still requires further study. Apart of the secretion of the pro-inflammatory cytokines, SD affects production of brain-derived neurotrophic factor (BDNF) while release of BDNF from astrocytes appears a key contributor to mood disorders. If and how the activation of NLRP3 inflammasome following SD affects the level of BDNF remains unknown. Antidepressant fluoxetine acts through astroglial 5-hydroxytryptamine receptor 2B (5-HT_2B_); these receptors are also related to the sleep-wake cycle. Contribution of leptin to MDD has been discovered recently, although the mechanistic links between leptin and the depressive-like behaviors has not been revealed. In this study, we discovered: (i) that activation of NLRP3 inflammasome was involved in the depressive-like behaviors induced by SD; (ii) decrease in BDNF following SD required the activation of NLRP3 inflammasomes; (iii) leptin augmented the anti-depressive action of fluoxetine through an increase in expression of astrocytic 5-HT_2B_ receptors. We suggest that decrease in BDNF by the activated NLRP3 inflammasomes in astrocytes is the key pathological event of the depressive-like behaviors induced by SD, while the combined treatment with fluoxetine and leptin improves therapeutic outcome for the depression induced by SD.

## Introduction

Sleep occupies almost one third of our life and it is necessary for human survival. Sleep loss results in various pathological manifestations, such as cognitive impairments (1), mood disorders (2), the increased circulating levels of inflammatory cytokines (3) and the dysfunction of the metabolite clearance from brain (4). Prolonged sleep deprivation or chronic sleep abnormalities are risk factors for the major depressive disorders (MDD) (5), whereas sleep disorders appear as a key symptom of MDD (6). However, the underlying mechanisms connecting sleep deprivation and MDD remain unclear.

Sleep deprivation (SD) can aggravate various pathological processes including neuroinflammation. Previously we have demonstrated that 6 h daily SD for 3 weeks triggers the activation of nucleotide-binding domain and leucine-rich repeat protein-3 (NLRP3) inflammasomes, and increases astroglial secretion of IL-1β and IL-18 (7). In addition, SD increases serum levels of brain-derived neurotrophic factor (BDNF) (8), which is related to sleep, cognition and learning (9–11), The BDNF released from astrocytes is also known to be associated with mood disorders (10). Both neuroinflamamtory (12) and the neurotrophin (10) hypothesis of MDD have been considered recently, albeit the links between inflammasome activation or BDNF secretion to the depressive symptoms induced by SD are unknown.

There is mounting evidence suggesting astrocytic abnormality in the major depression (13, 14), we previously reports the changes in the expression of 5-hydroxytryptamine 2B (5-HT_2B_) receptors in astrocytes following chronic mild stress (CMS) (15). The antidepressant effects of fluoxetine, a serotonin specific reuptake inhibitor (SSRIs), depend on astroglial 5-HT_2B_ receptors (16). We found that fluoxetine suppresses the activation of NLRP3 inflammasomes triggered by SD in astrocytes (7). The 5-HT_2B_ receptors are associated with regulation of sleep-wake cycle, as selective blockade of 5-HT_2B_ receptors increases the phase of wakefulness (14), reflecting the role of serotonin (5-HT) as the neurotransmitter contributing to physiological and pathological regulation of sleep-wake cycle (17). However, the role of 5-HT_2B_ receptors in the emergence of depressive-like behaviors induced by SD unknown. Recently, the therapeutic potential of leptin in the context of MDD has been discovered (18), although leptin efficacy in treating depressive-like behaviors following by SD has not been studied. The serum level of leptin is increased in the depressed women with normal weight with sleep disturbances, however the level of leptin is not changed in the overweight women (19).

In this work, we studied the mechanisms of leptin-induced facilitation of effects of fluoxetine on the depressive-like behaviors induced by SD and linked this effect to the increased expression of 5-HT_2B_ receptors in astrocytes.

## Methods And Materials

### Animals

Male C57BL/6 mice and FVB/N-Tg(GFAPGFP)14Mes/J (GFAP-GFP; #003257, RRID:IMSR_JAX:003257) mice were purchased from the Jackson Laboratory (Bar Harbor, ME, USA). As described previously (7), the mice were around 3 months old (~25 g) and were kept in standard housing conditions with food and water freely available. All operations were carried out in accordance with the USA National Institutes of Health's Guide for the Care and Use of Laboratory Animals (NIH Publications No. 8023) and its 1978 revision, and all experimental protocols were approved by the Institutional Animal Care and Use Committee of China Medical University.

### Materials

Most chemicals, including fluoxetine (fluoxetine hydrochloride; F132), SB204741 [*N*-(1-Methyl-1H-5-indolyl)-*N*′-(3-methyl-5-isothiazolyl)urea; S0693], U0126 [1,4-Diamino-2,3-dicyano-1,4-bis(o-aminophenylmercapto)butadiene monoethanolate; U120], LY294002 [2-(4-Morpholinyl)-8-phenyl-1(4H)-benzopyran-4-one hydrochloride; L9908], and a primary antibody raised against β-actin (A5441) were purchased from Sigma (MO, USA). Other primary antibodies were acquired from Millipore (MA, USA). Horseradish peroxidase-conjugated secondary antibodies were purchased from Santa Cruz Biotechnology (CA, USA). MCC950 (CP-456773; HY-12815) was purchased from MedChem Express (Shanghai, China). WP1066 [(E)-3(6-bromopyridin-2-yl)-2-cyano-N-(S0-1phenylethyl)acrylamide] was from EMD Chemicals (San Diego, CA, USA). Recombinant murine leptin (obesity protein; 450-31) was obtained from Preprotech Inc. (Jiangsu, China). BDNF enzyme-linked immunosorbent assay (ELISA) kit was from R&D Systems (MN, USA).

### Sleep Deprivation (SD) and Drug Treatment

FVB/N-Tg(GFAPGFP)14Mes/J mice were used for SD models. SD was induced by “gentle handling” according to standard protocols (20), including the introduction of new objects into the cage or gentle touching with a brush to keep the mice awake. As described previously (7), SD was induced for 6 h, from 7 a.m. to 1 p.m. During the treatment of SD, the mice were offered food and water *ad libitum*. Animals in the sham group were kept undisturbed in a separate room with the same light/dark cycle. The mice were treated with sham or SD stimulation for 3 weeks. During the third week, MCC950 (50 mg/kg for intraperitoneal injection), SB204741 (0.5 μmM in 2 μL for intracerebroventricular (ICV) infusion), U0126 (10 μM in 2 μL for ICV infusion), LY294002 (10 μM in 2 μL for ICV infusion), or vehicle (artificial cerebrospinal fluid, ACSF) was injected each day, then fluoxetine (10 mg/kg/day), leptin (4 mg/kg/day) or PBS (phosphate buffered saline) used as control group was injected intraperitoneally.

### Dissociation and Fluorescence-Activated Cell Sorting (FACS)

FVB/N-Tg(GFAPGFP)14Mes/J mice were used for collecting astrocytes. A purified astrocyte suspension isolated from the cortex and hippocampus was prepared as described previously (7). Briefly, tissue from two transgenic mice were pooled for one sample. Wavelengths of GFP excitation and emission were 488 and 530/30 nm, respectively. The purity of the sorted astrocytes has been confirmed in our previous study (21) by checking mRNA expression of cell markers of astrocytes, neurons and oligodendrocytes.

### Primary Culture of Astrocytes

Astrocytes were cultured according to our previous description (22, 23). In brief, primary cultures of mouse astrocytes were prepared from the cerebral hemispheres of newborn C57BL/6 mice. Cells were grown in Dulbecco's modified Eagle's medium (DMEM) with 7.5 mM glucose. For the entire third week, 0.25 mM dibutyryl cyclic AMP (dBcAMP) was included in the medium. All dishes of primary cultured astrocytes were randomly separated into different experimental groups.

### RNA Interference

As described previously (24), the cultured astrocytes were incubated in DMEM without serum for half day before transfection. A transfection solution containing 2 μl oligofectamine (Promega, Madison, WI, USA), 40 μl Opti-MEMI, and 2.5 μl siRNA (666 ng) was added to the culture in every well for 8 h. In the siRNA-negative control cultures, transfection solution without siRNA was added. Thereafter, DMEM with three times serum was added to the cultures. The siRNA duplex chains of leptin receptor (LepR), 5-HT_2B_ receptor (5-HT_2B_R) and *c-fos* were purchased from Santa Cruz Biotechnology (CA, USA).

### Behavior Tests

Twenty-four mice were randomly separated into different groups, and every mouse was scheduled for tests, and the test sequences were randomly assigned to avoid interference from different tests.

#### Tail Suspension Test

The tail suspension test is a behavioral despair-based test. As described previously (25), every mouse was suspended by its tail around 2 cm from the tip. Behavior was recorded for 6 min. The duration of immobility was calculated by an observer blinded to the treatment groups.

#### Forced Swimming Test

The forced swimming test is also a behavior despair-based test. Briefly, each mouse was trained to swim 15 min on the first day and was placed into a glass cylinder that contained 30 cm deep water (25 ± 1°C) for 6 min on the second day. The time of immobility was calculated during the last 4 min period which followed 2 min of habituation (25).

#### Sucrose Preference Test

The sucrose preference test is a reward-based test and a measure of anhedonia, as previously described by our group (15, 26). In brief, after 12 h of food and water deprivation, the mice were provided with two pre-weighed bottles, including one bottle that contained 2.5% sucrose solution and a second bottle filled with water, for 2 h. The percentage preference was calculated according to the following formula: % preference = [sucrose intake/(sucrose + water intake)] × 100%.

### Western Blotting

Western blotting was performed as previously described (23, 25). The protein content was determined by using BSA as the standard. Samples containing 50 μg protein were added into 10% polyacrylamide slab gels. After protein transfer to nitrocellulose membranes, the membranes were blocked by 5% skimmed milk powder in TBS-T for 2 h and then incubated with the primary antibody for 2 h at room temperature. After washing of the membrane, the specific binding was detected by goat-anti-rabbit or goat-anti-mouse horseradish peroxidase-conjugated secondary antibodies. Staining was visualized with ECL detection reagents, and images were acquired with an electrophoresis gel imaging analysis system. Band density was measured in Windows AlphaEase FC 32-bit software.

### Enzyme-Linked Immunosorbent Assay (ELISA)

According to our previously described (25, 27), BDNF level in the sorted astrocytes from GFAP-GFP transgenic mice or in the cultured astrocytes was measured via a total BDNF immunoassay kit methods. In brief, the measurements were performed according to the manufacturer's protocol. Samples were collected in pyrogen/endotoxin-free tubes. The lower limit of detection was <10 pg/ml.

### Quantitative PCR (qPCR)

Total RNA was reverse transcribed, and PCR amplification was performed with a Robocycler thermocycler, as our previously described (28). In brief, the relative quantities of the transcripts were assessed using 5-fold serial dilutions of RT product (200 ng). The RNA quantities were normalized to glyceraldehyde 3-phosphate dehydrogenase (GAPDH) before calculation of the relative expression of 5-HT_2B_R, c-fos, and LepR. Values were first calculated as the ratio of the relative expression of 5-HT_2B_R and GAPDH, then the values were normalized by control group.

### Statistical Analysis

Differences between multiple groups with one or two variables were evaluated by one-way or two-way analysis of variance (ANOVA) followed by Fisher's least significant difference (LSD) or a Tukey-Kramer *post-hoc* multiple comparison test for unequal replications using GraphPad Prism 5 software (GraphPad Software Inc., La Jolla, CA). Sample size was not predetermined by formal power calculation, and no samples or data were excluded from the analysis. All statistical data in the text are expressed as the mean ± SEM; the level of significance was set at *p* < 0.05.

## Results

### SD-Induced Depressive-Like Behaviors Depends On the Activation of NLRP3 Inflammasome

**Figure 1:**
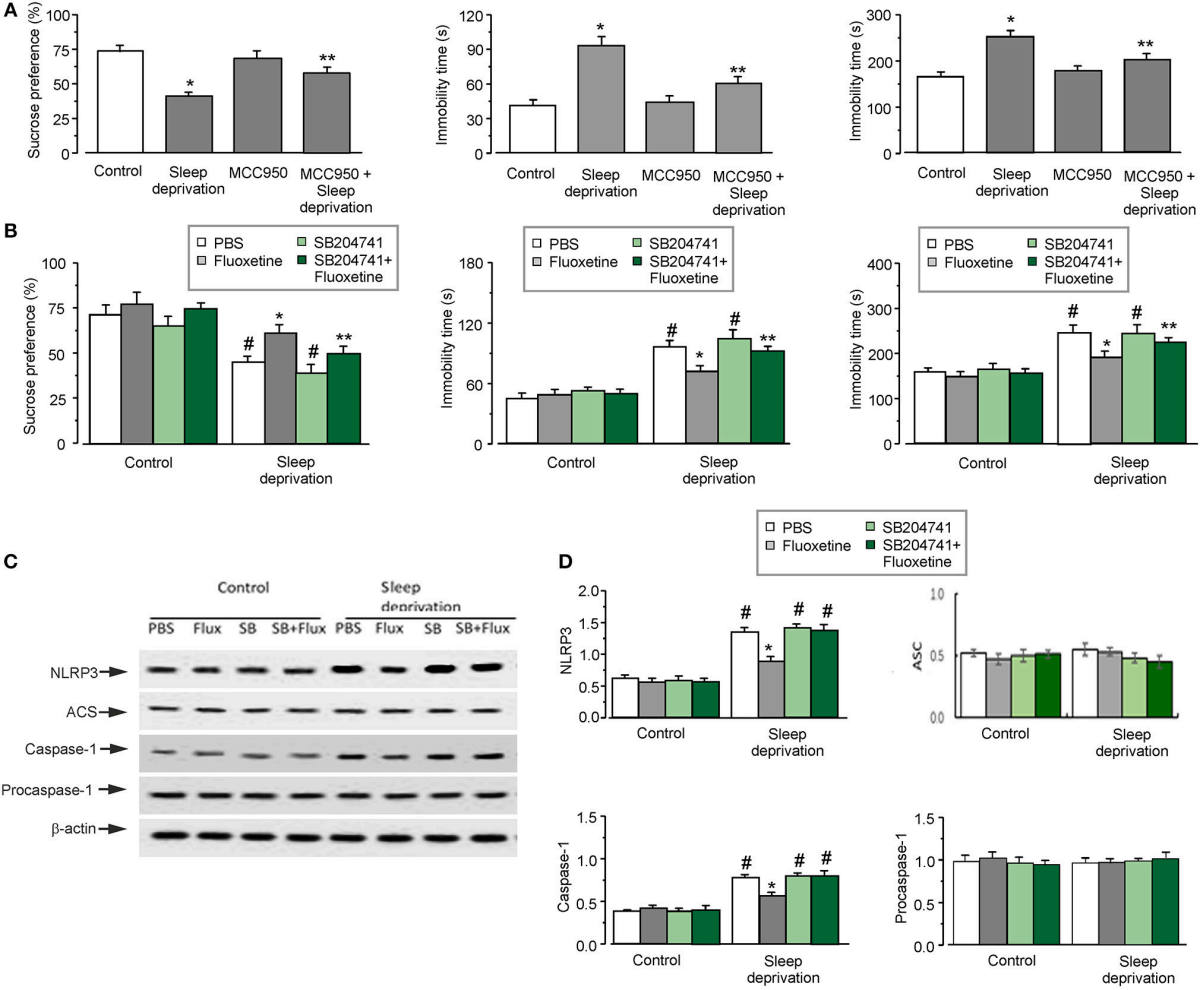
The depressive-like behaviors and the activation of NLRP3 inflammasomes induced by SD. **(A–D)** The GFAP-GFP transgenic mice were treated with sham (Control) or exposed to SD for 3 weeks, in the final week the mice were injected with or without MCC950 (a NLRP3 inhibitor), fluoxetine, 5-HT_2B_ receptors antagonist (SB204741; SB) or SB plus fluoxetine. The percentage of sucrose preference was measured (the left panels of **A** and **B**), the time of immobility were recorded in force-swimming test (the middle panels of **A** and **B**) and tail suspension test (the right panels of **A** and **B**), the values are expressed as mean ± SEM, *n* = 12. ^*^p < 0.05, statistically significant difference compared with any other group **(A)**; ^**^p < 0.05, statistically significant difference compared with control and SD groups **(A)**; ^*^p < 0.05, statistically significant difference compared with any other group **(B)**; ^**^p < 0.05, statistically significant difference compared with PBS and SD groups treated with sham (Control) or SD **(B)**; ^#^*p* < 0.05, statistically significant difference compared with any other group except for each other **(B)**. The astrocytes sorted from GFAP-GFP mice were collected, representative blots for the expression of NLRP3, ASC, caspase-1, procaspase-1 was shown in C1. Average protein levels were quantified as the ratio between the protein and β-actin, respectively, *n* = 6 **(D)**. ^*^p < 0.05, statistically significant difference compared with any other group **(D)**; ^#^*p* < 0.05, statistically significant difference compared with any other group except for each other **(D)**.

Three weeks of SD triggered depressive-like behaviors, as shown in Figures 1A,B. Exposure to SD decreased the percentage of sucrose preference by 44 ± 3.5% (*n* = 12) of control group (the left panel of Figure 1A). Meanwhile, 3-weeks of SD significantly elevated the immobility time by 126 ± 7.3 and 53 ± 9.5% (*n* = 12) of control group, in the forced swimming and tail suspension tests, respectively (the middle and right panels of Figure 1A). The depressive-like behaviors induced by SD required the activation of NLRP3 inflammasomes. Treatment with MCC950 (an inhibitor of NLRP3 inflammasomes) alleviated the depressive-like behaviors induced by SD: the percentage of sucrose preference was significantly increased by about 40 ± 4.2% (*n* = 12) compared with SD group (the left panel of Figure 1A), and the immobility time was reduced to 64 ± 6.3 or 80 ± 10.7% (*n* = 12) of SD group in the forced swimming or tail suspension test, respectively (the middle and right panels of Figure 1A).

### Fluoxetine Alleviation of SD-Induced Depressive-Like Behaviors and SD-Activated NLRP3 Inflammasomes Are Mediated by 5-HT_2B_ Receptor

Antidepressant fluoxetine effectively remedied the depressive-like behaviors induced by SD, as shown in Figure 1B. This effect of fluoxetine on depressive-like behaviors required the involvement of 5-HT_2B_ receptor. Treatment with SB204741 (an antagonist of 5-HT_2B_ receptor) inhibited effects of fluoxetine in behavioral tests: the pre-treatment with SB204741 decreased sucrose preference to the 73 ± 3.3% (*n* = 12) of the fluoxetine group (the left panel of Figure 1B), significantly prolonged the immobility time by 75 ± 6.1% (*n* = 12) in forced swimming test (the middle panel of Figure 1B), and by 35 ± 8.3% (*n* = 12) in tail suspension test compared with fluoxetine treated-SD group (the right panel of Figure 1B). However, sole treatment with SB204741 did not affect results of behaviors tests compared with neither control nor SD groups.

As have been shown previously, fluoxetine inhibits the activation of NLRP3 inflammasomes in astrocytes, which was induced by 3 weeks of SD of 3 weeks (7). Here, we further corroborated that inhibition of fluoxetine effect on NLRP3 inflammasome was mediated by 5-HT_2B_ receptor. In astrocytes isolated from transgenic mice fluoxetine reduced the protein level of NLRP3 and caspase-1 to 35 ± 4.3 and 72 ± 2.9% (*n* = 6) of SD group, respectively (Figure 1D). However, the pretreatment with SB204741 eliminated effects of fluoxetine on the expression of NLRP3 and caspase-1 in SD models (Figure 1D). Meanwhile, SD with or without fluoxetine had no effects on the protein level of ASC or procaspase-1 (Figure 1D).

### SD Decreases Astrocytic Level of BDNF via Activating NLRP3 Inflammasomes

**Figure 2:**
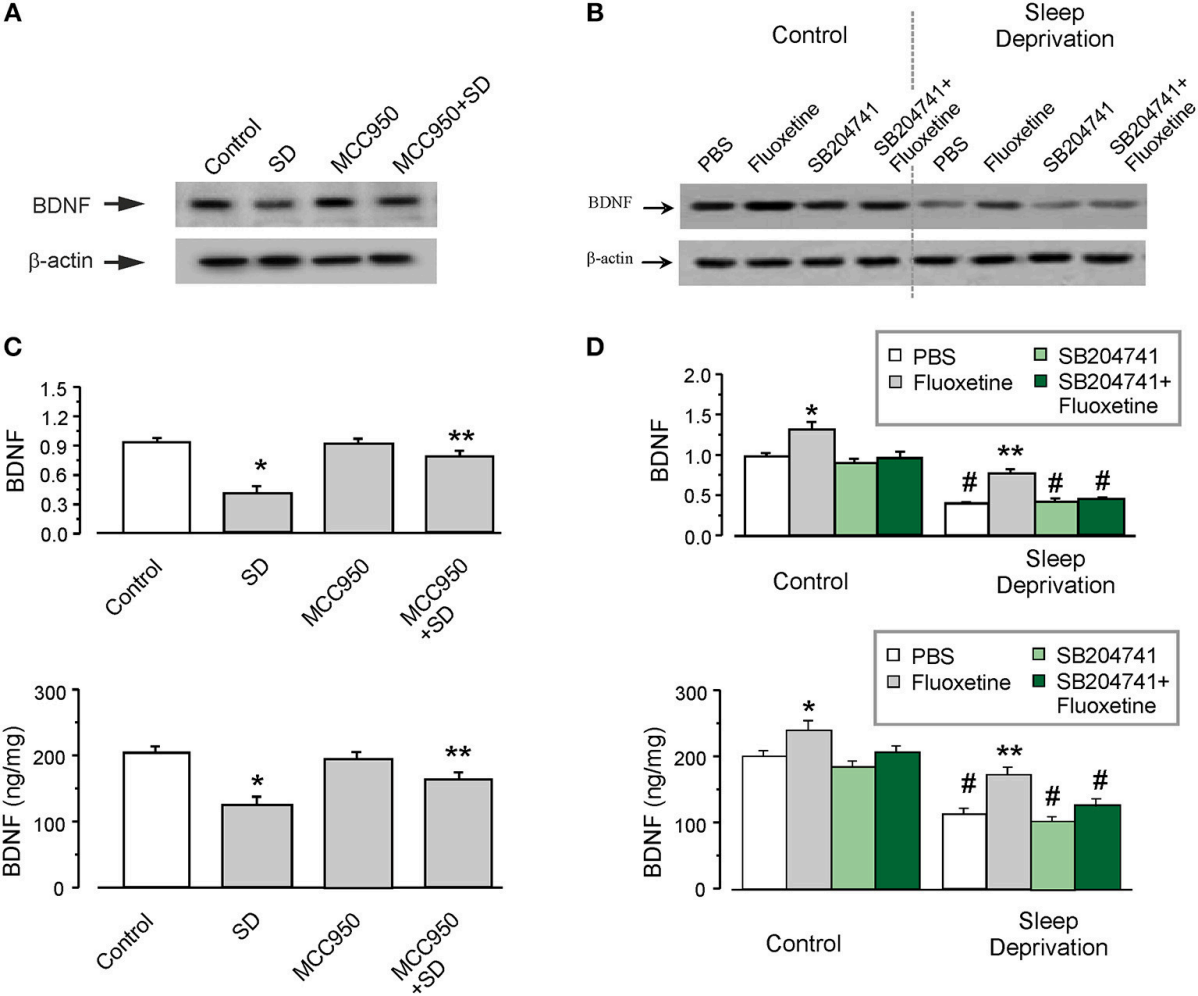
The protein level of BDNF decreased by SD. **(A–D)** The GFAP-GFP transgenic mice were treated with sham (Control) or exposed to SD for 3 weeks, in the final week the mice were intraperitoneally injected with or without MCC950, fluoxetine, 5-HT_2B_ receptors antagonist SB204741 (SB) or SB plus fluoxetine. The astrocytes sorted from GFAP-GFP mice were collected, representative blots for the expression of BDNF was shown in **(A,C)**. Average BDNF level was quantified as the ratio between the BDNF and β-actin, *n* = 6 (the upper panels of **B** and **D**). Meanwhile, the BDNF level was also measured via ELISA, *n* = 6 (the lower panels of **B** and **D**). ^*^p < 0.05, statistically significant difference compared with any other group **(B)**; ^**^p < 0.05, statistically significant difference compared with control and SD groups **(B)**; ^*^p < 0.05, statistically significant difference compared with any other group **(D)**; ^**^p < 0.05, statistically significant difference compared with fluoxetine group treated with sham (Control) and the other three groups treated with SD **(D)**; ^#^*p* < 0.05, statistically significant difference compared with any other group except for each other **(D)**.

The level of BDNF was significantly decreased in the astrocytes isolated from SD-treated transgenic mice, as shown in Figures 2A,B. Exposure to SD suppressed the protein expression of BDNF to 43 ± 3.3% (*n* = 6) of control group measured by western blotting (the upper panel of Figure 2B), the level of BDNF measured by ELISA was decreased by 39 ± 4.3% (*n* = 6), as compared with control group (the lower panel of Figure 2B). Furthermore, MCC950 (the inhibitor of NLRP3 inflammasomes) partly reversed this suppressive effect of SD on the level of BDNF (Figures 2A,B). In the isolated astrocytes, the protein expression of BDNF was elevated by 90 ± 2.7% (*n* = 6; the upper panel of Figure 2B), whereas the release of BDNF was increased by 30 ± 3.9% (*n* = 6; the lower panel of Figure 2B), as compared with SD group.

### The Level of BDNF Elevated by Fluoxetine Requires 5-HT_2B_ Receptor

As shown in Figure 2C, fluoxetine increased the expression of BDNF by 92 ± 4.2 or 36 ± 5.7% (*n* = 6), with or without SD treatment, respectively. Pretreatment with SB204741 (an antagonist of 5-HT_2B_ receptors), completely abolished the effect of fluoxetine on BDNF. Similarly, as compared with PBS group exposed to SD in animals, fluoxetine increased the level of BDNF by 50 ± 7.5% (*n* = 6), but after the pretreatment with SB204741, the elevated BDNF by fluoxetine was decreased by 10 ± 7.2% (*n* = 6; Figure 2D).

### The Signaling Cascade Contributing to Fluoxetine-Depended Decrease of Astrocytic BDNF

**Figure 3:**
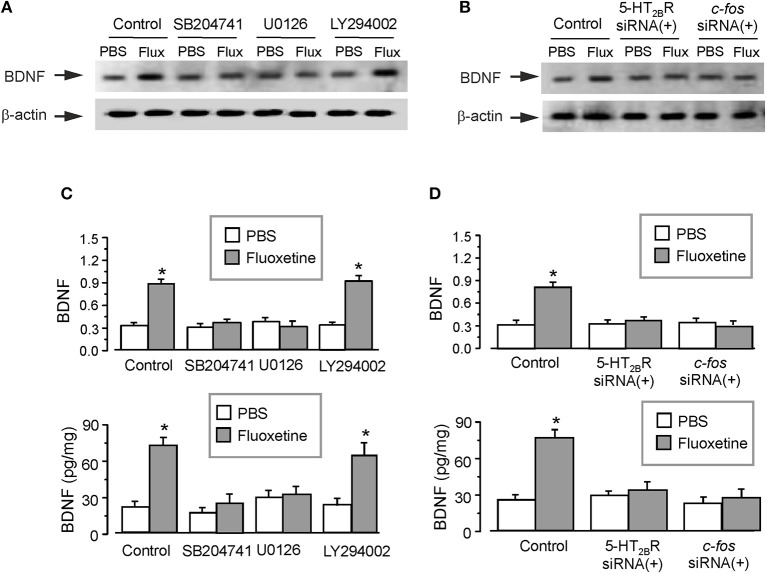
The regulation of fluoxetine on the level of BDNF in primary culture astrocytes. In the primary cultured astrocytes, after the pretreatment with or without 200 nM SB204741 (SB), 10 μM U0126 or 10 μM LY294002 **(A,C)**, or after RNA interfering the expression of 5-HT_2B_ receptors or cfos with siRNA duplex **(B,D)**, then the astrocytes were treated with PBS or fluoxetine for 1 week. Representative blots for the expression of BDNF was shown in **(A,B)**. Average BDNF level was quantified as the ratio between the BDNF and β-actin, *n* = 6 (the upper panels of **C** and **D**). Meanwhile, the average BDNF level was also measured via ELISA, *n* = 6 (the lower panels of **C** and **D**). ^*^p < 0.05, statistically significant difference compared with any other group except for each other **(B)**; ^*^p < 0.05, statistically significant difference compared with any other group **(D)**.

Administration of fluoxetine to the primary cultured astrocytes increased the expression of BDNF by 180 ± 5.1% (*n* = 6; Figure 3A). Similarly to *in vivo* experiments, SB204741 completely abolished the fluoxetine induced increase in BDNF expression (by 60 ± 4.2% (*n* = 6) of fluoxetine group). Pretreatment with U0126 (a selective inhibitor of MEK) completely abolished the fluoxetine-induced increase in expression of BDNF by 66 ± 4.7% of fluoxetine group (*n* = 6; Figure 3A). However, after the pretreatment with LY294002 [a selective inhibitor of phosphatidylinositol-3-kinase (PI3K)], fluoxetine still significantly increased the expression of BDNF by 176 ± 5.9% (*n* = 6) of control (PBS) group (Figure 3A). As shown in the lower panel of Figure 3C, the level of BDNF measured by ELISA had a similar tendency as the expression of BDNF measured by western blotting. SB204741 and U0126 dramatically abolished the fluoxetine induced increase in the level of BDNF by 65 ± 5.2 and 57 ± 4.7% (*n* = 6) of fluoxetine group, respectively. After pretreatment with LY294002, fluoxetine still increased the level of BDNF by 171 ± 6.2% (*n* = 6) of PBS group (the lower panel of Figure 3C).

Meanwhile, after treatment with interfering RNA of 5-HT_2B_ receptor, the protein level of BDNF increased by fluoxetine was suppressed to 111 ± 4.5 or 117 ± 6.9% (*n* = 6) of PBS group, as shown in Figures 3B,D. We also found that transcription factor *c-fos* was required for the fluoxetine induction of the expression of BDNF. Pretreatment with the siRNA duplex chains of *c-fos* eliminated differences between PBS group and fluoxetine group (Figures 3B,D). The relative expression of 5-HT_2B_R and c-fos were shown in Supplementary Figures 1A,B.

### Leptin Increases the Expression of 5-HT_2B_ Receptor on Astrocytes

**Figure 4:**
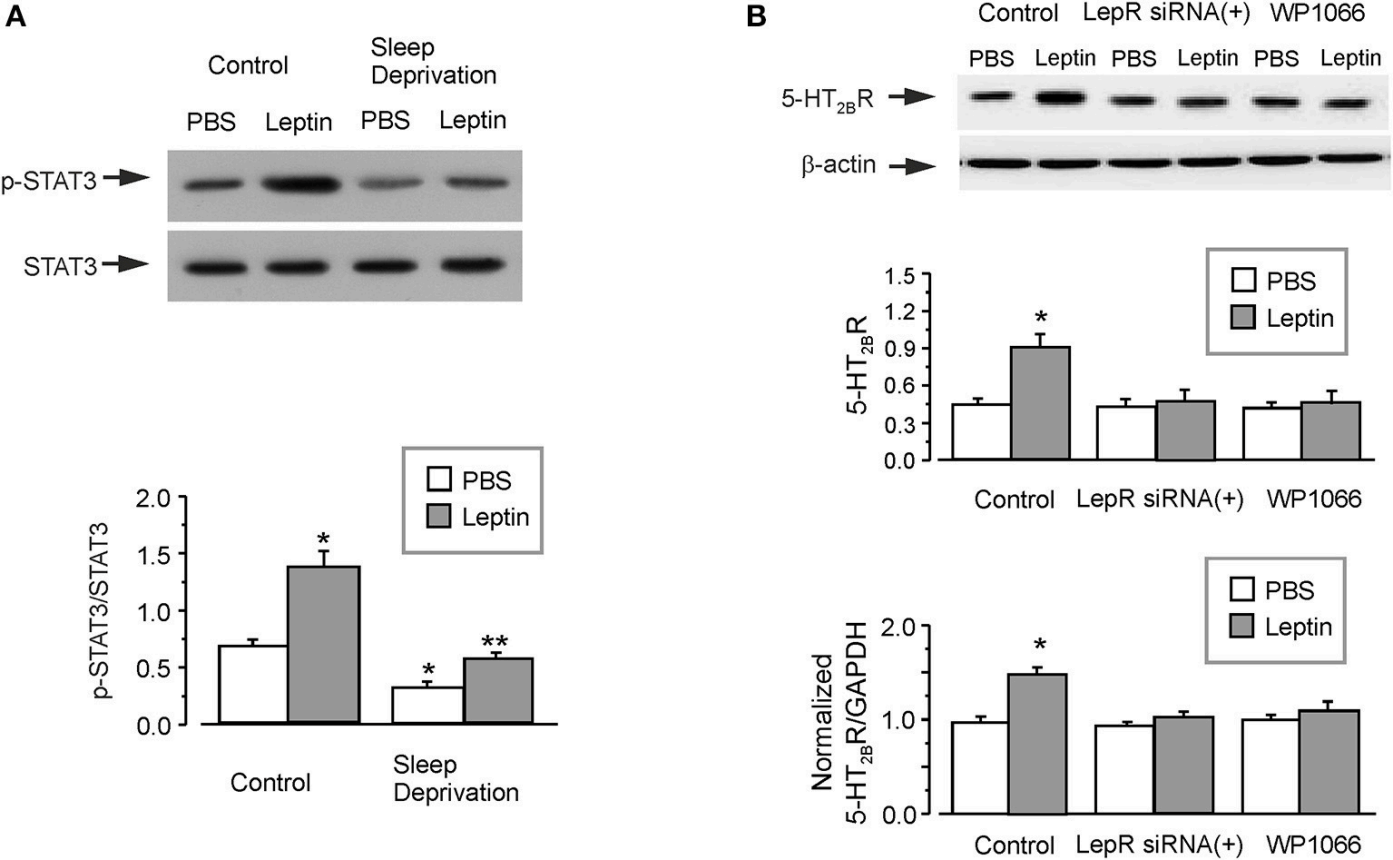
The regulation of leptin on the expression of 5-HT_2B_ receptors. The GFAP-GFP transgenic mice were treated with sham (Control) or exposed to SD for 3 weeks, in the final week the mice were injected with or without leptin **(A)**. Representative blots for the expression of p-STAT3 was shown in A1. The astrocytes sorted from GFAP-GFP mice were collected by FACS. The average phosphorylation level of STAT3 was quantified as the ratio between the p-STAT3 and STAT3, *n* = 6 **(A)**. ^*^p < 0.05, statistically significant difference compared with any other group **(A)**; ^**^p < 0.05, statistically significant difference compared with leptin group treated with sham (Control) and PBS group treated with SD **(A)**. After RNA interfering the expression of leptin receptors with siRNA duplex or 3 μM WP1066, an inhibitor of STAT3 **(B)**, then the primary cultured astrocytes were treated with PBS or leptin for 1 week. Representative blots for the expression of 5-HT_2B_ receptors was shown in B1. The average expression of 5-HT_2B_ receptors was quantified as the ratio between the 5-HT_2B_ receptors and β-actin, *n* = 6 (the upper panel of **B**). The mRNA expression of 5-HT_2B_ receptors was shown in **(C)**, the relative expression ratios of 5-HT_2B_R/GAPDH were normalized by the control group. Data represent mean ± SEM, *n* = 6. ^*^p < 0.05, statistically significant difference compared with any other group **(A,B)**.

The administration of leptin significantly induced the phosphorylation of STAT3 by about two times in the isolated astrocytes from GFAP-GFP mice (Figure 4A). However, in the SD-treated group, the ratio of p-STAT3/STAT3 was decreased to 46 ± 3.2% (*n* = 6) of control group (Figure 4A). Leptin re-elevated the phosphorylation of STAT3 decreased by SD to 177 ± 3.5% (*n* = 6) of SD-treated PBS group, but it was still only 80 ± 3.3% (*n* = 6) of PBS group without SD treatment (Figure 4A).

In the primary cultured astrocytes, treatment with leptin significantly increased the protein and mRNA expression of 5-HT_2B_ receptors by 100 ± 5.5 and 52 ± 4.7% (*n* = 6), respectively (Figure 4B). Treatment with RNA interfering with the expression of leptin receptors with siRNA duplex chains, completely the abolished effect of leptin on the expression of 5-HT_2B_ receptors. Pretreatment with WP1066 (an inhibitor of JAK2/STAT3) similarly decreases the protein and mRNA expression of 5-HT_2B_ receptors to the level of PBS group (Figure 4B). The relative expression of LepR was shown in Supplementary Figure 1C.

### Leptin Enhances the Function of Fluoxetine via the Increased 5-HT_2B_ Receptor

**Figure 5:**
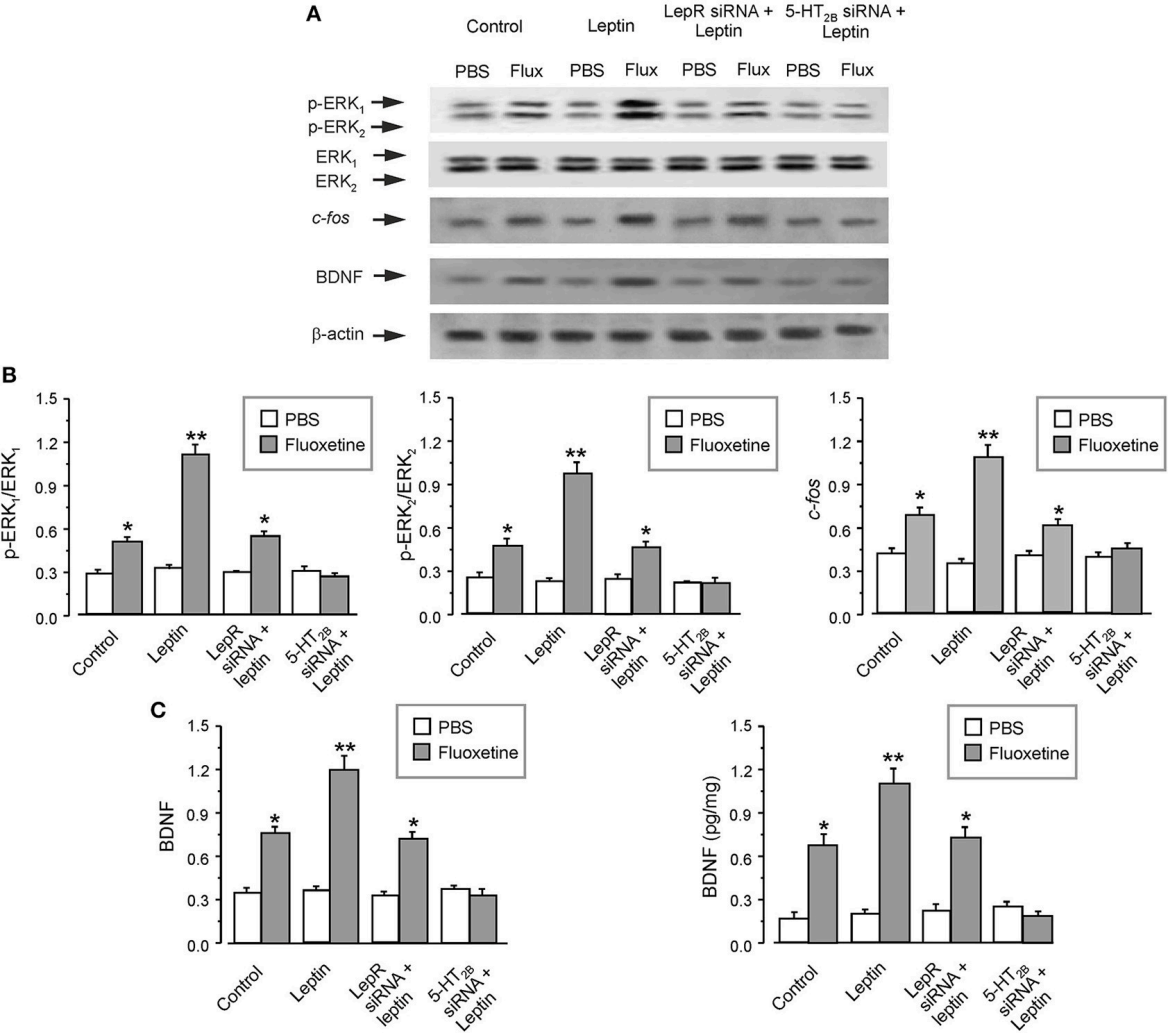
The regulation of leptin on the effects of fluoxetine in astrocytes. After RNA interfering the expression of leptin receptors or cfos with siRNA duplex, the primary cultured astrocytes were pre-treated with PBS (Control) or leptin, then the cells were treated with PBS or fluoxetine for 30 min to measure the phosphorylation of ERK_1/2_ or for 1 week to check the expression of the other proteins **(A–C)**. Representative blots for the level of p-ERK_1/2_, cfos and BDNF were shown in **(A)**. The average phosphorylation of ERK1 and ERK2 was quantified as the ratio between the p-ERK_1_ and ERK_1_ or p-ERK_2_ and ERK_2_, *n* = 6 (the left and middle panels of **B**), the average expression of cfos and BDNF were normalized by β-actin, *n* = 6 (the right panel of **B** and the left panel of **C**). The average BDNF level measured via ELISA was shown in **(B)**, *n* = 6. ^*^p < 0.05, statistically significant difference compared with any other group except for each other **(B,C)**; ^**^p < 0.05, statistically significant difference compared with any other group **(B,C)**.

After pretreatment of the primary cultured astrocytes with leptin, fluoxetine significantly increased the phosphorylation of ERK_1/2_ by 120 ± 7.2 and 106 ± 7.7% (*n* = 6), and enhanced the protein expression of *c-fos* or BDNF by 57 ± 6.5 or 57 ± 7.5% (*n* = 6), as compared with fluoxetine group (Figures 5A–C). The effect of leptin was mediated through leptin receptors, because after RNA interfering the expression of leptin receptors, leptin lost its ability to augment effects of fluoxetine on expression of p-ERK_1/2_, *c-fos* and BDNF (Figures 5A–C). At the same time after suppressing the expression of 5-HT_2B_ receptors, the effects of fluoxetine and leptin on the level of p-ERK_1/2_, *c-fos*, and BDNF were all abolished (Figures 5A–C).

### Leptin Augments Effects of Fluoxetine on the Depressive-Like Behaviors Induced by SD

**Figure 6:**
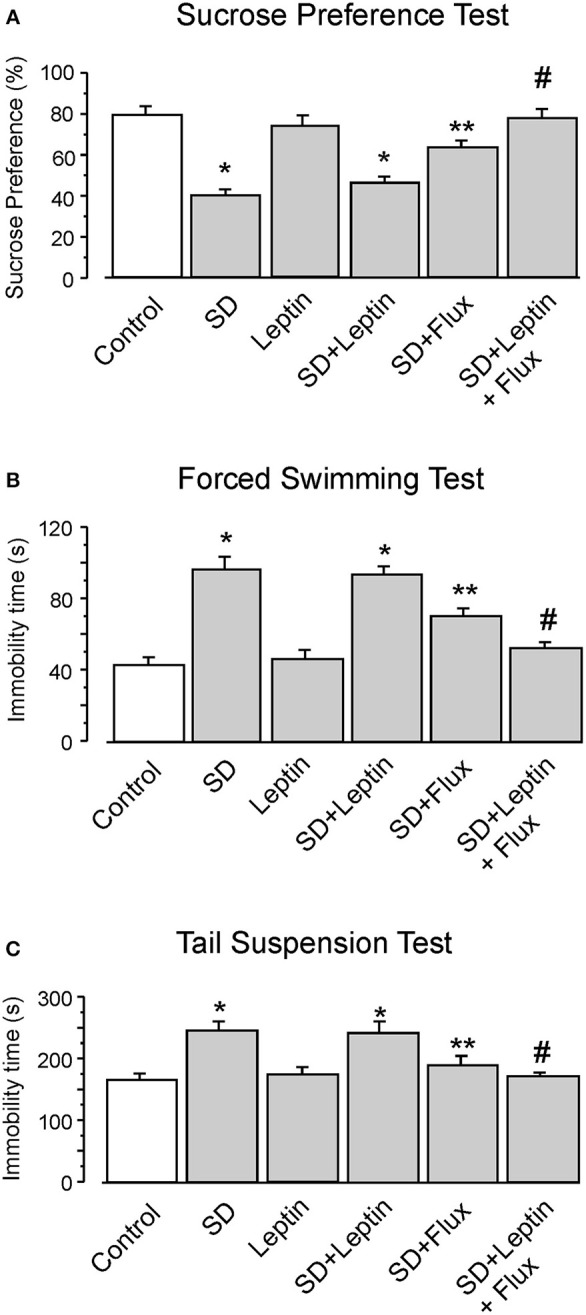
The GFAP-GFP transgenic mice were treated with sham (Control) or exposed to SD for 3 weeks, in the final week the mice were intraperitoneally injected with PBS, leptin, fluoxetine or leptin plus fluoxetine. The percentage of sucrose preference was measured **(A)**, the time of immobility were recorded in force-swimming test **(B)** and tail suspension test **(C)**, the values are expressed as mean ± SEM, *n* = 12. ^*^p < 0.05, statistically significant difference compared with any other group except for each other **(A–C)**; ^**^p < 0.05, statistically significant difference compared with any other group **(A–C)**; ^#^*p* < 0.05, statistically significant difference compared with SD, SD plus leptin and SD plus fluoxetine groups **(A–C)**.

In experiments *in vivo*, leptin augments effects of fluoxetine on the depressive-like behaviors. In sucrose preference test, SD decreased the sucrose uptake ratio by 49 ± 2.9% (*n* = 12), the administration of leptin alone had no effect. However, while fluoxetine increased this ratio by 59 ± 3.1% (*n* = 12) of SD group, treatment with leptin and fluoxetine combined increased the sucrose preference by 92 ± 5.7% (*n* = 12) of SD group, and by 21 ± 3.3% (*n* = 12) of SD plus fluoxetine group (Figure 6A). Similarly, in forced swimming test, leptin did not change the immobility time increased by SD, whereas treatment with leptin and fluoxetine decreased the immobility time to 54 ± 3.7% (*n* = 12) of SD group, and to 73 ± 3.1% (*n* = 12) of SD plus fluoxetine group (Figure 6B). Similar effect of leptin in tail suspension test is shown in Figure 6C, the immobility time of leptin plus fluoxetine in SD-treated group was decreased by 31 ± 2.9% (*n* = 12) in SD group, and by 12 ± 2.2% (*n* = 12) in SD plus fluoxetine group.

## Discussion

**Figure 7:**
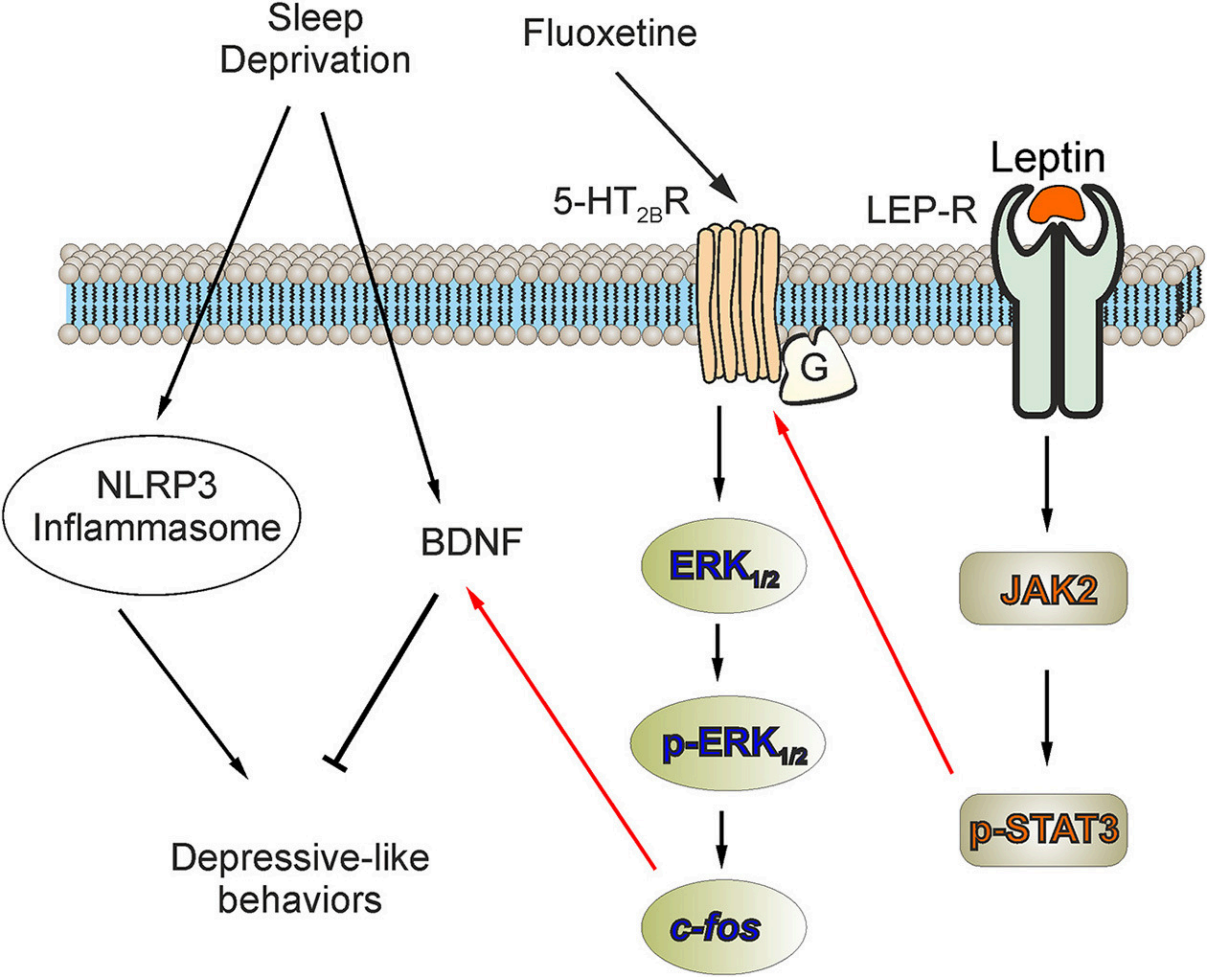
The facilitation of leptin on the improvement of fluoxetine for the depressive-like behaviors induced by SD. The activation of NLRP3 inflammasomes or the elimination of BDNF plays an important role in the depressive-like behaviors induced by SD. In our previous research, we reported that antidepressant fluoxetine could decrease the activation of NLRP3 inflammasomes triggered by SD. In this study, we discover that the activation of NLRP3 inflammasomes is involved in the elimination of BDNF in SD-treated mice. Meanwhile, fluoxetine increases the expression of BDNF via 5-HT_2B_ receptors. In primary cultured astrocytes, the expression of BDNF elevated by fluoxetine also requires the phosphorylation of ERK_1/2_ and the up-regulation of *c-fos*. Moreover, the pretreatment with leptin significantly increase the expression of 5-HT_2B_ receptors via LepR/JAK2/STAT3 pathway in astrocytes, so the improvement of fluoxetine for the level of BDNF and the depressive-like behaviors are obviously enhanced.

In this study, we discovered that activation of NLRP3 inflammasomes contributes to the development of the depressive-like behaviors induced by SD; the NLRP3 inflammasome also was associated to the decreased level of BDNF. The antidepressant fluoxetine triggers (through activation of 5-HT_2B_ receptors) the phosphorylation of ERK_1/2_ and increases the downstream expression of *c-fos*, which in turn increases the expression of BDNF in astrocytes. At the same time, leptin increases the expression of 5-HT_2B_ receptors via LepR/JAK2/STAT3 pathway in astrocytes, and fluoxetine could be more effective to elevate the level of BDNF and improve the depressive-like behaviors via the increased 5-HT_2B_ receptors induced by leptin (Figure 7).

The pathological mechanism of MDD is related to the activation of NLRP3 inflammasomes and the suppression of BDNF secretion (10, 12). For the first time, we show that SD decreases the level of BDNF and triggers the depressive-like behaviors via activating NLRP3 inflammations in astrocytes. Previously, we have discovered that SD activates NLRP3 inflammasomes and augments production of IL1β/18 in astrocytes, while fluoxetine obliterates neuroinflammation and neuronal apoptosis induced by SD (7). Sleep disturbance is often associated with stress-related mental disorders (29), but the potential mechanism linking these two diseases remains unknown. In the long term, sleep disturbance and MDD are both related to the decreased BDNF, whereas a part of SD-treatment induce a fast increase in BDNF serum level within hours, which has antidepressive effects (29), In this study, we demonstrate, that activated NLRP3 inflammasomes contribute to the SD-induced decrease in astrocytic BDNF levels. Although we did not measure the effect of astrocytic NLRP3 on integral or neuronal BDNF, the suppression of NLRP3 significantly decreased CUMS-induced inflammatory cytokines (caspase-1 and IL-1β) and apoptosis in hippocampus (30). And CUMS-induced the down-regulated BDNF and the activation of NLRP3 inflammasome in hippocampus or cortex could be both reversed in neuroligin3 (NLGN3) knockout mice (31).

Leptin is a 16 kDa hormone associated with regulation of energy balance and appetite (32); with growing evidence indicating its role in the MDD; it remains however a degree of controversy. Low CSF levels of leptin were detected in female suicide attempters with MDD (33), however, the serum leptin levels were not significantly changed in women with postpartum depression (34). The administration of leptin can produce a dose-dependent alleviation of depressive behaviors triggered by chronic stress and measured by forced swimming test or tail suspension test (35). But, the serum level of leptin in SD participants is 9.43 ± 8.87 ng/ml, which has no significant difference from the serum level of leptin in non-sleep-deprived persons (36). According to our results, the sole treatment with leptin does not improve the depressive like behaviors induced by SD in forced swimming or tail suspension test. In leptin receptors (LepR) knock-out mice, the anti-depressive-like behavioral effects of fluoxetine were unaffected (37). Our present study demonstrates that leptin enhances the antidepressive effects of fluoxetine through an increase of the expression of 5-HT_2B_ receptors in astrocytes.

Targeting astroglial 5-HT_2B_ receptors is an important part of therapy of depression (14). The therapeutic potential of fluoxetine and other serotonin-specific re-uptake inhibitors (SSRIs) is mediated, in part, through direct stimulation of astroglial 5-HT_2B_ receptors (16). In this work, we only checked the effects of fluoxetine on the increased expression of 5-HT_2B_ receptors induced by leptin, but the other SSRIs may play more antidepressive roles via the increased 5-HT_2B_ receptors. We demonstrated previously that acute treatment with fluoxetine induces the transactivation of epidermal growth factor (EGF) receptors (EGFR) and the phosphorylation of ERK_1/2_ by stimulating 5-HT_2B_ receptors in astrocytes (22). Furthermore, chronic treatment of astrocytes with fluoxetine increases the expression of *c-fos* through the 5-HT_2B_ receptors-EGFR-ERK_1/2_ pathway; this subsequently regulates expression of proteins, such as for example cPLA_2a_ and caveolin-1 (38, 39). Here we show that the decrease of astrocytic BDNF following SD is reversed by fluoxetine acting through *c-fos* induced by 5-HT_2B_ receptors-mediated ERK_1/2_ phosphorylation. At the same time leptin enhances the anti-depressive effects of fluoxetine through increasing the expression of 5-HT_2B_ in astrocytes under SD-condition.

In the clinic, sleep disorders and MDD are common pathologies; patients with sleep disturbances have a higher risk of the occurrence of MDD. To summarize our study, we found that chronic SD triggers the depressive-like behaviors by stimulating the activation of NLRP3 inflammasomes and by decreasing the expression of BDNF in astrocytes. We further found that fluoxetine alleviates the depressive-like behaviors and rescues decreased BDNF release induced by SD by stimulating 5-HT_2B_ receptors. Leptin enhances fluoxetine effects by increasing the expression of astroglial 5-HT_2B_ receptors. Thus, the decrease in astrocytic BDNF release induced by the activated NLRP3 inflammasomes is the key pathological mechanism of the depressive-like behaviors induced by SD, while the co-treatment with fluoxetine and leptin can provide an effective therapy for the depressive-like behaviors induced by SD by regulating expression of astroglial 5-HT_2B_ receptors.

## Author Contributions

MX and BL designed the experiments. XL and SSL built the animal models and analyzed the data. XL operated cell culture. ZL and SL performed other experiments. AV and BL wrote the paper.

### Conflict of Interest Statement

The authors declare that the research was conducted in the absence of any commercial or financial relationships that could be construed as a potential conflict of interest.

## Supplementary Material

Supplementary Figure 1 The mRNA expressions after RNA interfering. The expression of 5-HT_2B_ receptors (5-HT_2B_R), c-fos and leptin receptors (LepR) with negative control or siRNA duplex, the relative expression ratios of 5-HT_2B_R/GAPDH, c-fos/GAPDH and LepR/GAPDH were normalized by the control group and shown in **(A–C)**. Data represent mean ± SEM, *n* = 6. ^*^*p* < 0.05, statistically significant difference compared with any other group.

## References

[1] Euston DR Steenland HW. Neuroscience. Memories–getting wired during sleep. Science (2014) 344:1087–8. 10.1126/science.1255649 24904140

[2] Benca RM Peterson MJ. Insomnia and depression. Sleep Med. (2008) 9:S3–9. 10.1016/S1389-9457(08)70010-8 18929317

[3] Shearer WT Reuben JM Mullington JM Price NJ Lee BN Smith EO. Soluble TNF-alpha receptor 1 and IL-6 plasma levels in humans subjected to the sleep deprivation model of spaceflight. J Allergy Clin Immunol. (2001) 107:165–70. 10.1067/mai.2001.112270 11150007

[4] Xie L Kang H Xu Q Chen MJ Liao Y Thiyagarajan M. Sleep drives metabolite clearance from the adult brain. Science (2013) 342:373–7. 10.1126/science.1241224 24136970 PMC3880190

[5] Alvaro PK Roberts RM Harris JK Bruni O. The direction of the relationship between symptoms of insomnia and psychiatric disorders in adolescents. J Affect Disord. (2017) 207:167–74. 10.1016/j.jad.2016.08.032 27723540

[6] Adrien J. Neurobiological bases for the relation between sleep and depression. Sleep Med Rev. (2002) 6:341–51. 10.1053/smrv.2001.0200 12531125

[7] Xia M Li X Yang L Ren J Sun G Qi S The ameliorative effect of fluoxetine on neuroinflammation induced by sleep deprivation. J Neurochem. (2017) 146:14272 10.1111/jnc.14272 29222907

[8] Giese M Beck J Brand S Muheim F Hemmeter U Hatzinger M. Fast BDNF serum level increase and diurnal BDNF oscillations are associated with therapeutic response after partial sleep deprivation. J Psychiatr Res. (2014) 59:1–7. 10.1016/j.jpsychires.2014.09.005 25258340

[9] Faraguna U Vyazovskiy VV Nelson AB Tononi G Cirelli C. A causal role for brain-derived neurotrophic factor in the homeostatic regulation of sleep. J Neurosci. (2008) 28:4088–95. 10.1523/JNEUROSCI.5510-07.2008 18400908 PMC2597531

[10] Duman RS Heninger GR Nestler EJ. A molecular and cellular theory of depression. Arch Gen Psychiatry (1997) 54:597–606. 10.1001/archpsyc.1997.01830190015002 9236543

[11] Duman RS Malberg J Nakagawa S D'Sa C. Neuronal plasticity and survival in mood disorders. Biol Psychiatry (2000) 48:732–9. 10.1016/S0006-3223(00)00935-5 11063970

[12] Alcocergómez E Cordero MD NLRP3 inflammasome: a new target in major depressive disorder. CNS Neurosci Ther. (2014) 20:294–5. 10.1111/cns.12230 24479787 PMC6493047

[13] Verkhratsky A Rodriguez JJ Steardo L. Astrogliopathology: a central element of neuropsychiatric diseases? Neuroscientist (2014) 20:576–88. 10.1177/1073858413510208 24301046

[14] Peng L Song D Li B Verkhratsky A Astroglial 5-HT_2B_ receptor in mood disorders. Expert Rev Neurother. (2018) 30:1–8. 10.1080/14737175.2018.1458612 29600729

[15] Dong L Li B Verkhratsky A Peng L. Cell type-specific *in vivo* expression of genes encoding signalling molecules in the brain in response to chronic mild stress and chronic treatment with fluoxetine. Psychopharmacology (2015) 232:2827–35. 10.1007/s00213-015-3921-2 25851944

[16] Peng L Gu L Li B Hertz L. Fluoxetine and all other SSRIs are 5-HT_2B_ agonists–importance for their therapeutic effects. Curr Neuropharmacol. (2014) 12:365–79. 10.2174/1570159X12666140828221720 25342944 PMC4207076

[17] Kantor S Jakus R Balogh B Benko A Bagdy G. Increased wakefulness, motor activity and decreased theta activity after blockade of the 5-HT_2B_ receptor by the subtype-selective antagonist SB-215505. Br J Pharmacol. (2004) 142:1332–42. 10.1038/sj.bjp.0705887 15265808 PMC1575194

[18] Ubani CC Zhang J. The role of adiposity in the relationship between serum leptin and severe major depressive episode. Psychiatry Res. (2015) 228:866–70. 10.1016/j.psychres.2015.05.009 26032460

[19] Häfner S Baumert J Emeny RT Lacruz ME Thorand B Herder C. Sleep disturbances and depressed mood: A harmful combination associated with increased leptin levels in women with normal weight. Biol Psychol. (2012) 89:163–9. 10.1016/j.biopsycho.2011.10.005 22020135

[20] Franken P Dijk DJ Tobler I Borbély AA. Sleep deprivation in rats: effects on EEG power spectra, vigilance states, and cortical temperature. Am J Physiol. (1991) 261:198–208. 10.1152/ajpregu.1991.261.1.R198 1858947

[21] Fu H Li B Hertz L Peng L. Contributions in astrocytes of SMIT1/2 and HMIT to myo-inositol uptake at different concentrations and pH. Neurochem Int. (2012) 61:187–94. 10.1016/j.neuint.2012.04.010 22564531

[22] Li B Zhang S Zhang H Nu W Cai L Hertz L. Fluoxetine-mediated 5-HT_2B_ receptor stimulation in astrocytes causes EGF receptor transactivation and ERK phosphorylation. Psychopharmacol (Berl). (2008) 201:443–58. 10.1007/s00213-008-1306-5 18758753

[23] Li B Zhang S Zhang H Hertz L Peng L. Fluoxetine affects GluK2 editing, glutamate-evoked Ca(2+) influx and extracellular signal-regulated kinase phosphorylation in mouse astrocytes. J Psychiatry Neurosci. (2011) 36:322–38. 10.1503/jpn.100094 21320410 PMC3163648

[24] Li B Ren J Yang L Li X Sun G Xia M. Lithium inhibits GSK3β activity via two different signaling pathways in neurons after spinal cord injury. Neurochem Res. (2018) 43:1–9. 10.1007/s11064-018-2488-9 29404840

[25] Xia M Yang L Sun G Qi S Li B. Mechanism of depression as a risk factor in the development of Alzheimer's disease: the function of AQP4 and the glymphatic system. Psychopharmacology (2016) 234:1–15. 10.1007/s00213-016-4473-9. 27837334

[26] Li B Dong L Wang B Cai L Jiang N Peng L. Cell type-specific gene expression and editing responses to chronic fluoxetine treatment in the *in vivo* mouse brain and their relevance for stress-induced anhedonia. Neurochem Res. (2012) 37:2480–95. 10.1007/s11064-012-0814-1 22711334

[27] Li B Qi S Sun G Yang L Han J Zhu Y. Leptin suppresses adenosine triphosphate-induced impairment of spinal cord astrocytes. J Neurosci Res. (2016) 94:924–35. 10.1002/jnr.23795 27316329

[28] Lundgaard I Li B Xie L Kang H Sanggaard S Haswell JDR. Direct neuronal glucose uptake heralds activity-dependent increases in cerebral metabolism. Nat Commun. (2015) 6:6807. 10.1038/ncomms7807 25904018 PMC4410436

[29] Schmitt K Holsboertrachsler E Eckert A. BDNF in sleep, insomnia, and sleep deprivation. Ann Med. (2016) 48:42–51. 10.3109/07853890.2015.1131327 26758201

[30] Zhang CY Zeng MJ Zhou LP Li YQ Zhao F Shang ZY. Baicalin exerts neuroprotective effects via inhibiting activation of GSK3β/NF-κB/NLRP3 signal pathway in a rat model of depression. Int Immunopharmacol. (2018) 64:175–82. 10.1016/j.intimp.2018.09.001 30195108

[31] Li ZQ Yan ZY Lan FJ Dong YQ Xiong Y. Suppression of NLRP3 inflammasome attenuates stress-induced depression-like behavior in NLGN3-deficient mice. Biochem Biophys Res Commun. (2018) 501:933–40. 10.1016/j.bbrc.2018.05.085 29775613

[32] Hukshorn C Saris W. Leptin and energy expenditure. Curr Opin Clin Nutr Metab Care (2004) 7:629–33. 10.1097/00075197-200411000-00007 15534430

[33] Westling S Ahrén B Träskmanbendz L Westrin A. Low CSF leptin in female suicide attempters with major depression. J Affect Disord. (2004) 81:41–8. 10.1016/j.jad.2003.07.002 15183598

[34] Yildiz G Senturk MB Yildiz P Cakmak Y Budak MS Cakar E. Serum serotonin, leptin, and adiponectin changes in women with postpartum depression: controlled study. Arch Gynecol Obstet. (2017) 295:853–8. 10.1007/s00404-017-4313-0 28224268

[35] Lu XY Kim CS Frazer A Zhang W. Leptin: a potential novel antidepressant. Proc Natl Acad Sci USA. (2006) 103:1593–8. 10.1073/pnas.0508901103 16423896 PMC1360555

[36] Abu-Samak MS Mohammad BA Abu-Taha MI Hasoun LZ Awwad SH. Associations between sleep deprivation and salivary testosterone levels in male university students: a prospective cohort study. Am J Mens Health (2018) 12:411–9. 10.1177/1557988317735412 29025356 PMC5818117

[37] Guo M Lu XY. Leptin receptor deficiency confers resistance to behavioral effects of fluoxetine and desipramine via separable substrates. Transl Psychiatry (2014) 4:e486. 10.1038/tp.2014.126 25463972 PMC4270309

[38] Li B Zhang S Li M Hertz L Peng L. Chronic treatment of astrocytes with therapeutically relevant fluoxetine concentrations enhances cPLA_2_ expression secondary to 5-HT_2B_-induced, transactivation-mediated ERK_1/2_ phosphorylation. Psychopharmacology (2009) 207:1–12. 10.1007/s00213-009-1631-3 19662385

[39] Li B Jia S Yue T Yang L Huang C Verkhratsky A. Biphasic regulation of Caveolin-1 gene expression by fluoxetine in astrocytes: opposite effects of PI3K/AKT and MAPK/ERK signaling pathways on c-fos. Front Cell Neurosci. (2017) 11:335. 10.3389/fncel.2017.00335 29163047 PMC5671492

