# Supplementary figures and images for: Leptin Increases Expression of 5-HT2B Receptors in Astrocytes Thus Enhancing Action of Fluoxetine on the Depressive Behavior Induced by Sleep Deprivation
Source: Front Psychiatry. 2019 Jan 7;9:734. doi: 10.3389/fpsyt.2018.00734 (PMC6330762; doi:10.3389/fpsyt.2018.00734)

**Supplementary Figure 1**


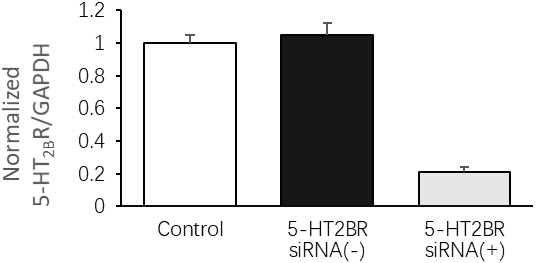


**A**

*****


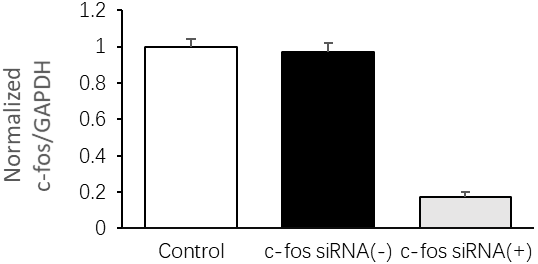


**B**

*****


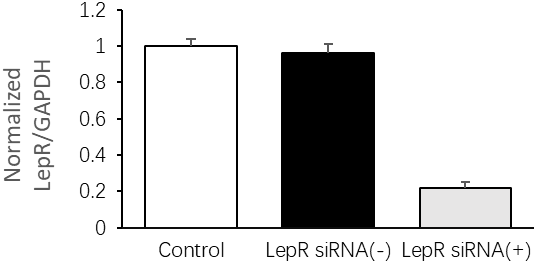


**C**

*****

Supplement: Supplementary Figure 1 — The mRNA expressions after RNA interfering. The expression of 5-HT2B receptors (5-HT2BR), c-fos and leptin receptors (LepR) with negative control or siRNA duplex, the relative expression ratios of 5-HT2BR/GAPDH, c-fos/GAPDH and LepR/GAPDH were normalized by the control group and shown in (A–C). Data represent mean ± SEM, n = 6. *p < 0.05, statistically significant difference compared with any other group. [file Data_Sheet_1.docx]
